# Biomass-Based Nanocomposites of Polydithioacetals Derived from Vanillin with Cellulose Nanocrystals: Synthesis, Thermomechanical and Reprocessing Properties

**DOI:** 10.3390/polym17131764

**Published:** 2025-06-26

**Authors:** Lei Li, Xibin Shen, Jianglu Teng, Bo Zhao, Sixun Zheng

**Affiliations:** 1 College of Chemistry and Chemical Engineering and the State Key Laboratory of Metal Matrix Composites, Shanghai Jiao Tong University, Shanghai 200240, China; polylli@sjtu.edu.cn (L.L.); 022110910063@sjtu.edu.cn (X.S.);; 2 Xinjiang Keli New Technology Development Co., Ltd., Karamay 834000, China

**Keywords:** cellulose nanocrystals, nanocomposites, nanoreinforcement, polydithioacetals, reprocessing, vanillin

## Abstract

Bio-based polydithioacetal nanocomposites were synthesized to address the critical need for materials that simultaneously achieve enhanced thermomechanical properties and excellent reprocessing capabilities. Using vanillin and cellulose nanocrystals (CNCs) as starting materials, linear polydithioacetals (PDTAs) were prepared via acid-catalyzed polycondensation of vanillin with various dithiols including 1,6-hexanedithiol, 1,10-decanedithiol, 3,6-dioxa-1,8-octanedithiol and 2,2′-thiodiethanethiol. These PDTAs were then crosslinked with a diepoxide (i.e., diglycidyl ether of bisphenol A, DGEBA) via the reaction of phenolic hydroxyl groups of PDTAs with epoxide groups of DGEBA. To create the nanocomposites, cellulose nanocrystals (CNCs) were surface-functionalized with thiol groups and then incorporated as the reinforcing nanofillers of the networks. The results of morphological observation showed that the fine dispersion of CNCs in the polymer matrix was attained. Owing to the incorporation of CNCs, the nanocomposites displayed improved thermomechanical properties. Compared to the network without CNCs, the nanocomposite containing 20 wt% CNCs exhibited an increase of more than tenfold in modulus and threefold in tensile strength. In addition, the nanocomposites exhibited excellent reprocessing properties, attributable to the dynamic exchange of dithioacetal bonds. This work presents a promising strategy for developing bio-based nanocomposites that have not only improved thermomechanical properties but also excellent reprocessing (or recycling) properties.

## 1. Introduction

In recent years, considerable interest has been attracted to the development of nanocomposites with desirable properties by taking advantage of a rich variety of biomasses [1,2,3]. This approach involves fabricating the materials through sustainable chemistries. The key aspects of these nanocomposites are twofold: (i) to explore the chemistries to generate the materials through the utilization of biomass feedstock and (ii) to endow the materials with reprocessability and recyclability. Derived from biomass, the compounds such as vanillin, cellulose, lignin, rosin acids and terpenes can be exploited through a series of effective reactions, depending on their chemical structure and functionality [4,5,6]. To achieve reprocessing (or recycling), investigators have recently explored the integration of dynamic chemical bonds (DCBs) into networks of polymers. Benefiting from the exchange of DCBs, the networks can be rearranged under external stimuli such as heat, light or pH. As a result, the materials can be self-healable and/or reprocessable [7,8,9,10,11,12]. In 2011, Leibler et al. [13] first demonstrated this strategy in anhydride-crosslinked epoxy networks. Under the catalysis of zinc acetate, the transesterification exchange of hydroxyether ester structural units was activated, thereby introducing dynamic behavior into the networks. It was found that the epoxy networks can be re-shuffled at elevated temperature while the thermomechanical properties of the epoxy networks were maintained at lower service temperature. Inspired by this concept, investigators have explored the use of other dynamic chemistries in a variety of polymeric networks, to endow the thermosets with thermal adaptability, including transesterification [14,15,16,17,18], disulfide exchange [19,20,21,22], imine exchange [23,24,25], silyl ether exchange [26,27,28], boronicester exchange [29,30,31,32,33], carbonate exchange [34] and thioacetal exchange [35,36,37,38,39,40,41,42,43,44]. Recently, the exchange of dithioacetal bonds has provoked considerable interest due to the simplicity of material processing. Dithioacetal and polydithioacetal bonds can be generated through base-catalyzed alkynyl-thiol Michael addition [45], acid-catalyzed aldehyde (or ketone)-thiol thioacetalization [39], acid-catalyzed acetal-thiol reaction [46] and acetal-thiol click-like reaction [35]. Owing to the high dynamicity, dithioacetal bonds have been integrated into polymeric networks to gain self-healing and reprocessing properties [35,36,37,38,39,40,41,42,43]. Du Prez et al. [45] reported the synthesis of dithioacetal-containing networks via the reactions of trithiol with activated alkynes. It was found that the thioacetal crosslinkages were robust but dynamic, imparting excellent reprocessing properties to the networks. Guo et al. [47] synthesized a dithioacetal-containing diacid (BTA), which was then used as a crosslinker of epoxidized natural rubber (ENR). It was found that the exchange of dithioacetal can be thermally activated, enabling the rearrangement of the network structures, i.e., the crosslinked rubbers displayed excellent reprocessability and adjustable mechanical properties. More recently, Cui et al. [48] reported the synthesis of the crosslinked polydithioacetal (PDTA) via solvent-free polycondensation of biomass benzaldehyde and tetra-thiol monomers at room temperature. Notably, in addition to their self-healing properties, these crosslinked PDTAs exhibited multiple modes of recyclability, including mechanical reprocessing, chemical recycling and monomer recycling.

Vanillin is a bio-derived aromatic aldehyde and extractable from lignin, which simultaneously carries a phenolic hydroxyl group [49,50,51,52,53]. By taking advantage of these highly reactive groups, vanillin can be integrated into networks of polymers with a variety of reactions. For instance, Zhang et al. [54] synthesized an aldehyde-epoxy monomer (VE) from vanillin, which was then mixed with DGEBA and pentaerythritol tetra(3-mercaptopropionate) to obtain epoxy networks containing dithioacetal linkages. Yuan et al. [55] reported the synthesis of the linear and cross-linked polydithioacetals (PDTAs) through thiol-aldehyde polycondensation of vanillin with polymercaptans. The cross-linked networks showcased excellent reprocessing and degradation properties. More recently, Yuan et al. [56] reported an efficient cross-linking strategy through the reaction of vanillin-derived monomer with 1,6-hexanedithiol. The dithioacetal linkages not only impart processability and oxidative degradability to the cross-linked epoxy resins but also enhance their stability in hydrolytic environments. Celluloses are a class of polysaccharides with β-D-glucopyranosyls as the repeating units. Derived from cellulose, cellulose nanocrystals (CNCs) possess a well-defined, rigid and fibrous nanostructure [57,58,59]. This feature enables CNCs to serve as effective nanoreinforcement to improve the properties of polymers, allowing the fabrication of sustainable materials [60,61,62]. Reinforcing bio-based PDTA networks is therefore of significant interest. To the best of our knowledge, however, there has been no previous report in this regard.

In this contribution, we report a new approach to access nanocomposites with biomass-derived compounds [i.e., vanillin and cellulose nanocrystals (CNCs)] as the feedstock. First, the polycondensations of vanillin with dithiols were carried out to afford polydithioacetals (PDTAs) with variable main-chain structures. Thereafter, these PDTAs were post-crosslinked with a commercial diepoxide (e.g., DGEBA) via the reaction of epoxide with phenolic hydroxyl groups. It is anticipated that the networks of PDTAs are reprocessable through the dynamic exchange of dithioacetal bonds. To further reinforce the networks, CNCs were incorporated to generate the nanocomposites. The goal of this work is twofold: (i) to demonstrate the generation of the networks of PDTAs with a commercial diepoxide (e.g., diglycidyl ether of bisphenol A) as the crosslinker; (ii) to reveal the reinforcement of PDTA networks with a biomass nanofiller (*viz.* CNCs).

## 2. Materials and Methodology

### 2.1. Materials

Vanillin, 1,6-hexanedithiol, 1,10-decanedithiol, 3,6-dioxa-1,8-octanedithiol, 2,2′-thiodiethanethiol, tetrabutylammonium bromide (TBAB), trifluoroacetic acid and 3-mercaptopropyltriethoxysilane were purchased from Shanghai Titan Scientific Co., Ltd., Shanghai, China. Diglycidyl ether of bisphenol A (DGEBA) with a quoted epoxide value of 0.51 mol/100 g was purchased from Nantong Xingchen Synthetic Material Co., Nantong, China. Cellulose nanocrystals (CNCs) were purchased from Science K Co., Beijing, China, with the sizes of approximately 500 nm in length and 10~20 nm in diameter. The organic solvents were purchased from Shanghai Titan Scientific Co., Ltd., China.

### 2.2. Synthesis of Polydithioacetals (PDTAs)

Linear polydithioacetals (PDTAs) were synthesized by following the approach of the literature [55] with slight modification. Typically, vanillin (4.560 g, 30 mmol) and 3,6-dioxa-1,8-octanedithiol (DI) (5.469 g, 30 mmol) were dissolved in methanol (10 mL), and then trifluoroacetic acid (0.1 M, 6 mL) was added; the reactions were carried out at 30 °C for 2 h. Notably, the polymerized products were gradually separated from the initial homogeneous mixtures with the polymerization proceeding. The crude polymers were isolated through removing the soluble components. The crude products were washed with methanol three times. After that, the products were dried in vacuo at 40 °C for 24 h; the yields were gravimetrically calculated. In all the cases, the yields as high as 90% were obtained. For PDTA-DI, the yield was 91%. ^1^H NMR (ppm, CDCl_3_): 7.10~6.78 (4H, -C_6_***H***_4_-), 5.24 (1H, -S-C***H***-S-), 4.14 (3H, -OC***H***_3_), 3.58~3.96 [4H, -C***H***_2_O-] and 2.78~3.12 (8H, -S-C***H***_2_C***H***_2_O-). GPC: *M*_n_ = 36,700 Da with *M*_w_/*M*_n_ = 1.36. By using various dithiols, the polydithioacetals (PDTAs) can be synthesized with various main-chain structures as summarized in Table 1.

### 2.3. Surface Functionalization of CNCs

To a flask containing a magnetic stirrer, CNCs (5.000 g) and toluene (100 mL) were charged. Under vigorous stirring, 3-mercaptopropyltriethoxysilane (20.000 g) was added. Thereafter, the mixture was heated to 80 °C, at which the reaction was performed for 24 h. The product was collected by filtration, washed three times with tetrahydrofuran and then dried in vacuo at 40 °C for 24 h. The product (i.e., the surface-functionalized CNCs) was obtained with a yield of 90%. FTIR (KBr, cm^−1^): 3345 (O-H), 2520 (S-H) and 817 (Si-O). TGA: the yield of residues of 3.55% at 800 °C.

### 2.4. Preparation of Nanocomposites of PDTAs with CNCs

Typically, DGEBA (1.656 g), the surface-functionalized CNCs (0.300 g) and 1,4-dioxane (10 mL) were added to a flask equipped with a magnetic stirrer. Triethylamine (5 mg) was added as a catalyst for the reaction between the thiol groups on CNCs and the epoxy groups of DGEBA. Heated to 60 °C, the mixture was maintained at this temperature for 10 h. Thereafter, the solution of PDTA-DI (4.044 g) dissolved in 1,4-dioxane (5 mL) and TBAB (0.180 g) was added. The reaction was then conducted at 120 °C for an additional 10 h. After the majority of the solvent was removed through rotary evaporation, the product was further dried in vacuo at 40 °C overnight to obtain the nanocomposite. By varying the loading of CNCs, the nanocomposites with variable compositions were obtained and denoted as *c*-PDTA-DI-CNC5, *c*-PDTA-DI-CNC10, *c*-PDTA-DI-CNC15 and *c*-PDTA-DI-CNC20, respectively, where the digits represent the mass fractions of CNCs.

## 3. Results and Discussion

### 3.1. Synthesis of PDTAs and Surface Functionalization of CNCs

**Scheme 1:**
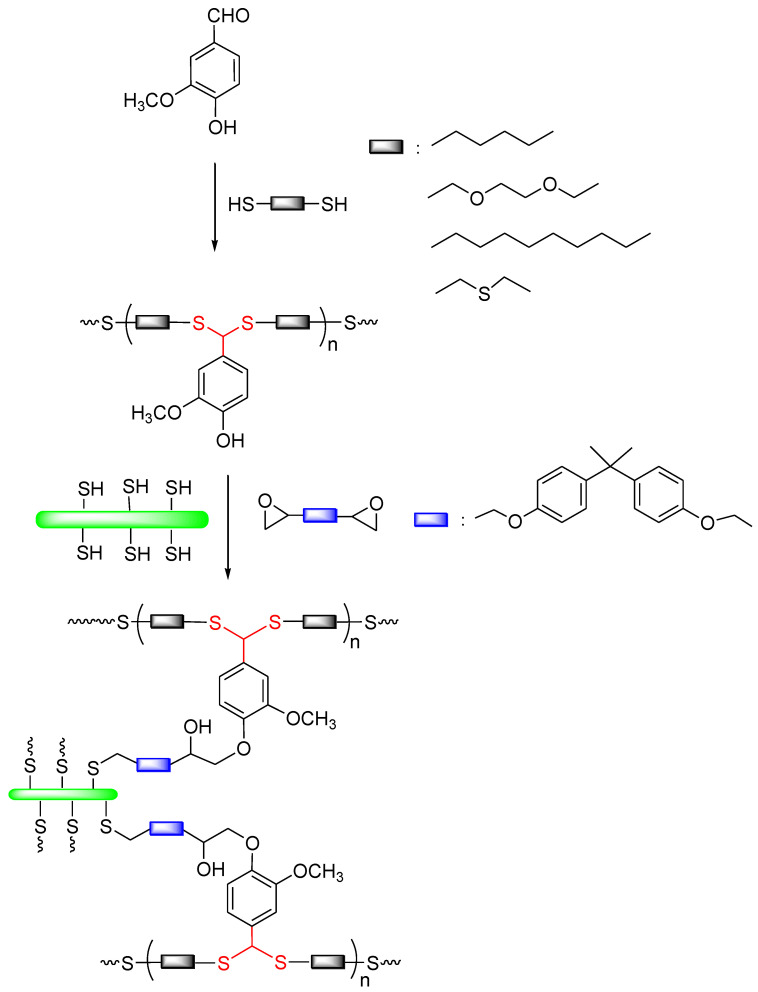
Synthesis of biomass-based nanocomposites.

**Figure 1:**
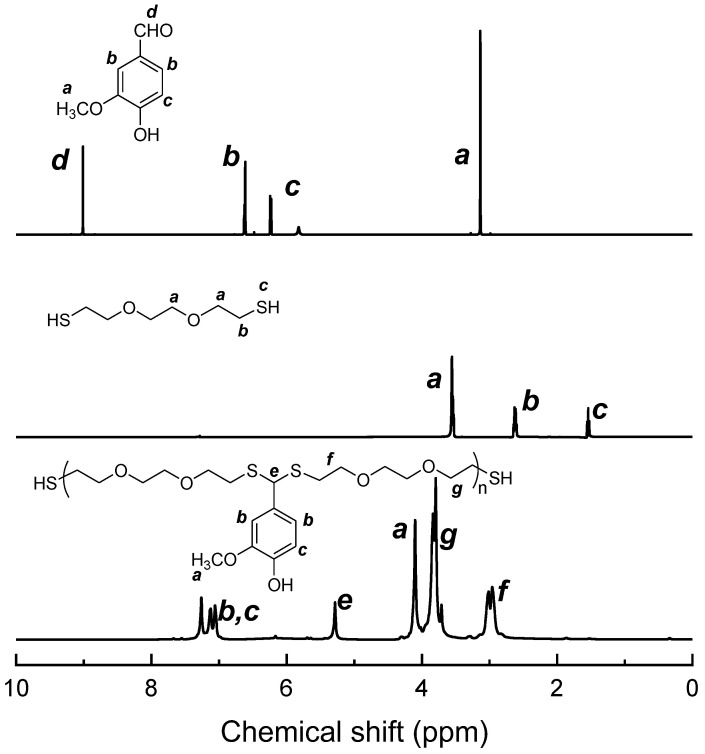
^1^H NMR spectra of vanillin, 3,6-dioxa-1,8-octanedithiol and PDTA-DI.

**Figure 2:**
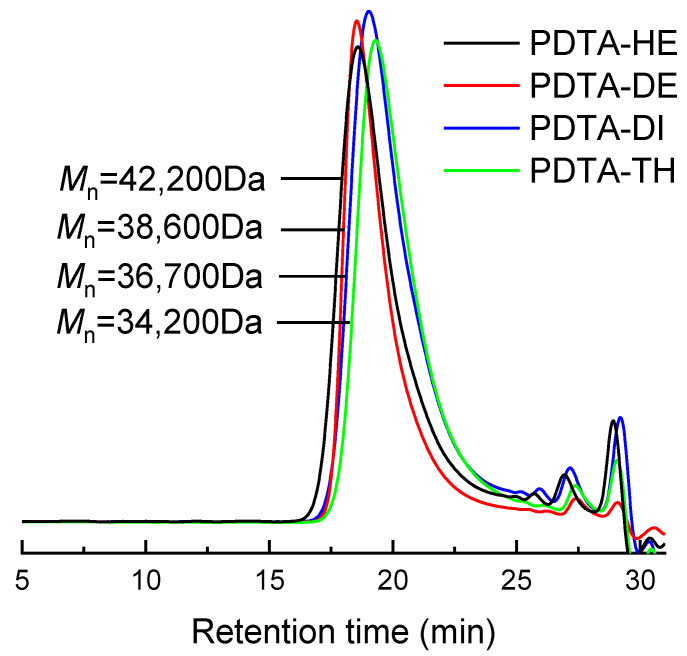
GPC profiles of PDTAs.

The route of synthesis for PDTAs is shown in Scheme 1. The polycondensations of vanillin with dithiols including 1,6-hexanedithiol (HE), 1,10-decanedithiol (DE), 2,2′-thiodiethanethiol (TH) and 3,6-dioxa-1,8-octanedithiol (DI) were carried out to synthesize linear polydithioacetals (PDTAs) with various main-chain structures as summarized in Table 1. Taking PDTA-DI for instance, the ^1^H NMR spectra are presented in Figure 1. For vanillin, the resonance of aldehyde, methoxy and phenyl protons were detected at 9.61, 3.16 and 6.2~6.6 ppm, respectively [55]. For 3,6-dioxa-1,8-octanedithiol, the resonance of methylene protons adjacent to the oxygen and sulfur atoms appeared at 3.56 and 3.21 ppm, whereas that of thiol protons was detected at 1.56 ppm. Upon polymerization, the peaks of resonance assignable to the aldehyde group of vanillin (9.61 ppm) and thiol groups of 3,6-dioxa-1,8-octanedithiol (1.56 ppm) fully disappeared. In the meantime, a new signal appeared at 5.24 ppm, corresponding to the methine protons of the newly formed dithioacetal (S-CH-S) linkage. The results of ^1^H NMR spectroscopy indicate that the polycondensation of vanillin with the diol was successfully performed. For all of these PDTAs derived from various dithiols, Fourier transform infrared (FTIR) spectroscopy was carried out as shown in Figure S1. In all of these cases, no infrared bands were detected at 1668 and 2566 cm^−1^. The former was assignable to the carbonyl group of aldehyde, whereas the latter was assignable to the S-H bonds of dithiols. The FTIR spectroscopy indicates the occurrence of condensation of aldehyde with thiol groups. At the same time, there appeared the band at 755 cm^−1^, assignable to the stretching vibration of C-S bonds, confirming the formation of dithioacetal linkages. For all of these PDTAs, the broad bands at 3415 cm^−1^ were detected, attributable to the stretching vibration of phenolic hydroxyl groups. All of these PDTAs were subjected to gel permeation chromatography (GPC); the as-recorded GPC chromatograms are shown in Figure 2. For all of these samples, the unimodal distribution of molecular weights was exhibited, and the number-average molecular weights were measured in the range of *M*_n_ = 3.40 × 10^4^~4.20 × 10^4^ Da with *M*_w_/*M*_n_ = 1.32~1.40. The sufficiently high *M*_n_ values indicate that the polycondensations were effectively conducted between vanillin and various dithiols.

The surface functionalization of cellulose nanocrystals (CNCs) was carried out via the reaction of the surface hydroxyl groups with thiol groups of 3-mercaptopropyltriethoxysilane. FTIR spectra of pristine and thiol-functionalized CNCs are shown in Figure S2. Both the pristine and surface-functionalized CNCs displayed the broad and intense bands in the range of 3000~4000 cm^−1^, assignable to the stretching vibration of hydroxyl groups. With the reaction with 3-mercaptopropyltriethoxysilane, there appeared two new bands at 817 and 2520 cm^−1^, resulting from the bending vibration of Si-O groups and the stretching vibration of thiol groups (S-H), respectively. The detection ofS-O bonds is a sign of the reaction of hydroxyl groups with ethoxy groups of 3-mercaptopropyltriethoxysilane, whereas the appearance of S-H bonds indicates that the surface of CNCs was successfully functionalized. To characterize the quantity of surface functionalization, the product was further subjected to thermogravimetric analysis (TGA). For comparison, the pristine CNCs were also measured under identical conditions. As shown in Figure S3, pristine CNCs were fully degraded and decomposed in an air atmosphere, whereas the surface-functionalized CNCs had the residues of degradation. It is proposed that the residues of degradation resulted from the organosilicon moieties (i.e., triethoxysilane), which were converted into silica due to the thermal oxidation of the sample at elevated temperature (i.e., 800 °C). Therefore, the quantity of 3-mercaptopropyltriethoxysilane (*A*) at the surface of CNCs can be calculated according to the following equation [60]:

(1) $$A=\frac{W_{1}-W_{2}}{M_{org}}\times 100\%$$

where *W*_1_ and *W*_2_ are the residues for 1 g of pristine CNCs and thiol-functionalized CNCs, and *M*_org_ was the mass of the organic components in the thiol-functionalized CNCs. In terms of the yield of degradation at 800 °C (*viz.* 3.55 wt%), the surface thiol group content was calculated to be 0.592 mmol × g^−1^.

### 3.2. Generation of Nanocomposites of PDTAs with CNCs

By taking advantage of the reaction of phenolic hydroxyl groups with epoxide groups, all of these linear PDTAs were crosslinked with a diepoxide (i.e., DGEBA) as the crosslinker. In all of these cases, the molar ratios of phenolic hydroxyl groups to epoxide groups were controlled to be 1:1; the crosslinked products were denoted as *c*-PDTA-HE, *c*-PDTA-DE, *c*-PDTA-TH and *c*-PDTA-DI, respectively, where the first letter “*c*” represents “crosslinking”, whereas the last two letters refer to the names of the corresponding dithiols. As shown in Figure S4, the FTIR spectra showed that the bands at 910 cm^−1^, assignable to epoxide groups, fully disappeared, indicating that the crosslinking reactions were complete. To assess the degree of crosslinking, the gel fractions of all of these *c*-PDTA networks were measured through the extraction tests as detailed in Supporting Information (SI). In all of these cases, the gel fractions were measured to be 94.6 % or higher (Figure S5), demonstrating that the crosslinking reactions of PDTAs with DGEBA were quite effective.

**Figure 3:**
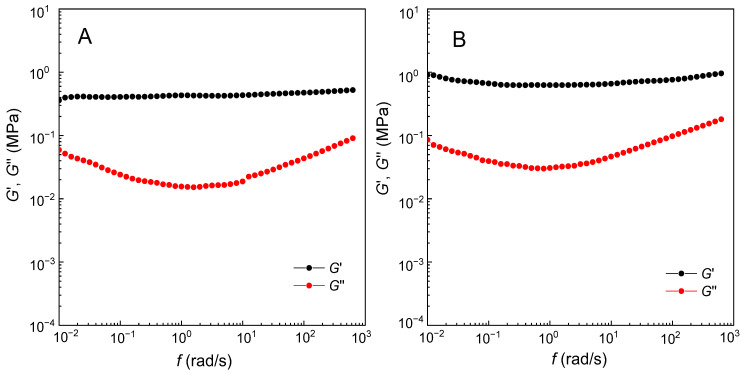
Rheological data of frequency sweeps of (**A**) *c*-PDTA-DI-CNC10 and (**B**) *c*-PDTA-DI-CNC20.

To facilitate the dispersion of CNCs in the matrices of *c*-PDTAs, the surfaces of CNCs were functionalized with thiol groups, which can also undergo the reaction with epoxide groups of DGEBA. To generate the nanocomposites, the surface-functionalized CNCs were first reacted with DGEBA, and then PDTAs were incorporated. In this work, the molar ratio of epoxide groups to the phenol groups of PDTA-DI was controlled to be 0.7:1, and the contents of CNCs were varied from 0 to 20 wt% to obtain the nanocomposites with variable compositions, which were named as “*c*-PDTA-DI-CNC*x*”, where *x* is the weight percentage of CNCs. To investigate the reinforcement of CNCs, the nanocomposites were subjected to rheological analysis with frequency sweeps. The rheological data are shown in Figure 3 and Figure S6. For all of these samples, the dynamic storage moduli (*G*′) were higher than the dynamic loss moduli (*G*″), demonstrating that the samples displayed solid-like behavior. The rheological analyses indicate that the network structures were generated in these composites. For all of these samples, notably, the *G*’ values remained nearly unchanged across the tested frequency range from 10^−2^ to 10^2^ rad × s^−1^, suggesting that at 100 °C the samples behaved as perfectly crosslinked rubbers. In addition, higher contents of CNCs led to higher *G*′ values, indicating that the CNCs served as the reinforcement of the *c*-PDTA-DI networks.

**Figure 4:**
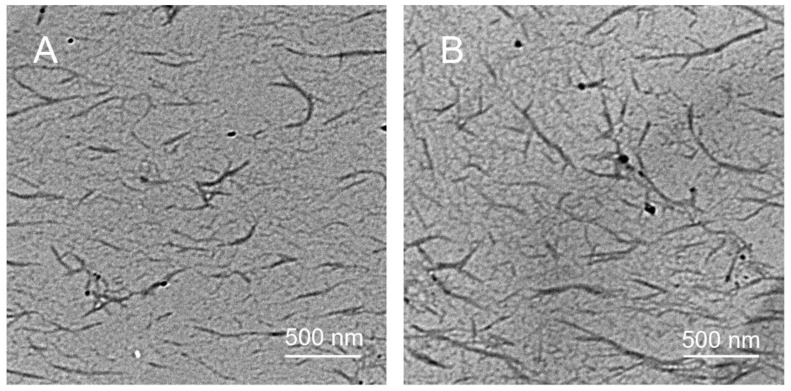
TEM images of (**A**) *c*-PDTA-DI-CNC10 and (**B**) *c*-PDTA-DI-CNC20.

To ascertain the dispersion of CNCs in the *c*-PDTA networks, the morphological structures of the nanocomposites were investigated with transmission electron microscopy (TEM). Representatively shown in Figure 4 are the TEM images of the nanocomposites of *c*-PDTA-DI with 10 and 20 wt% of CNCs. It is seen that the nanoscale heterogeneous morphologies are exhibited; the dark fibrous nano-objects were uniformly dispersed in the shallow continuous matrix. Given the significant difference in electron densities between CNCs and the *c*-PDTA-DI matrix, the dark fibrous features are attributed to CNCs, whereas the light matrix corresponds to the C-PDTA-DI networks. Notably, the fibrous CNCs with a length of 500 nm and a diameter of 10~20 nm are homogeneously dispersed in the *c*-PDTA-DI matrices. The fine dispersion of CNCs is attributable to the formation of the chemical linkages between *c*-PDTA-DI and CNCs. The chemical linkages were generated via (i) the reaction of the surface thiol groups of CNCs with epoxide groups of DGEBA and (ii) the reaction of phenolic groups of PDTA with epoxide groups as indicated in Scheme 1. It is the generation of chemical linkages that suppressed the aggregation of CNCs. The fine dispersion is critical for achieving the mechanical reinforcement of the materials.

### 3.3. Thermal and Mechanical Properties

**Figure 5:**
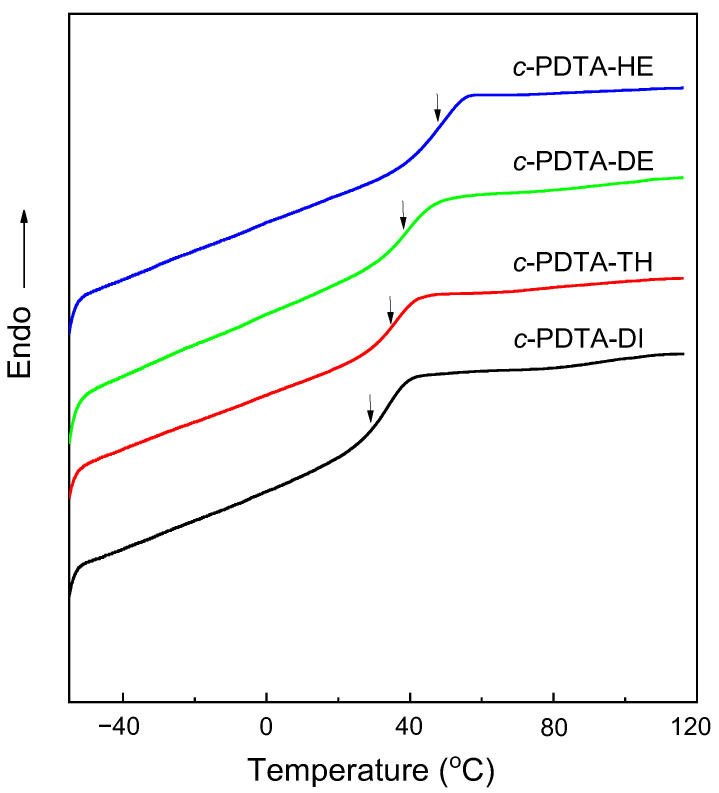
DSC curves of *c*-PDTA-HE, *c*-PDTA-DE, *c*-PDTA-TH and *c*-PDTA-DI.

**Figure 6:**
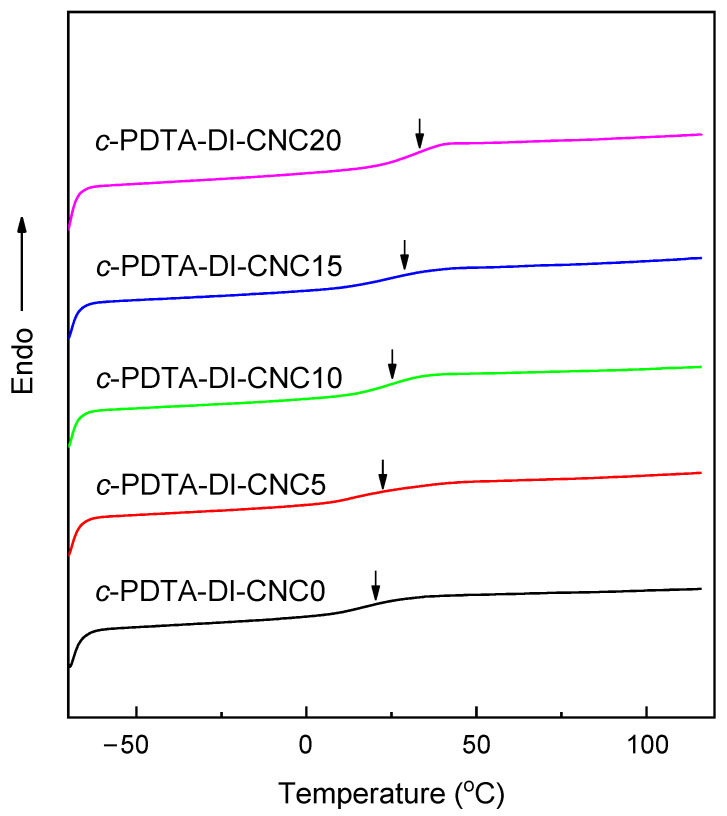
DSC curves of *c*-PDTA-DI-CNC nanocomposites.

The thermal and mechanical properties of the nanocomposites were investigated through differential scanning calorimetry (DSC). Shown in Figure 5 are the DSC curves of the control networks derived from various dithiols. The single glass transitions were displayed in all of these cases, indicating the homogeneity of the networks. Notably, the glass transition temperatures (*T*_g_’s) were quite dependent on the structures of the dithiols used. From *c*-PDTA-DI, *c*-PDTA-TH, *c*-PDTA-DE and *c*-PDTA-HE networks, the *T*_g_’s were measured to be 27.8, 33.6, 37.8 and 48.0 °C, respectively. The increase in *T*_g_’s is readily associated with the decrease in the molecular weights (or molecular sizes) of dithiols. The lower the molecular weights of dithiols, the higher the crosslinking densities of *c*-PDTA networks. As a result, *c*-PDTA-HE displayed the highest *T*_g_. The incorporation of CNCs also led to an increase in the *T*_g_’s. Taking the nanocomposites of *c*-PDTA-DI-CNCs, for instance, the DSC measurements were carried out, and DSC curves are shown in Figure 6. Notably, the *T*_g_’s increased with the increment of the contents of CNCs. For the *c*-PDTA-DI-CNC0 network, the *T*_g_ was 20.9 °C. The incorporation of 20 wt% of CNCs resulted in the *T*_g_ being enhanced to 32.7 °C. The increase in *T*_g_’s is attributable to the nanoreinforcement of the fibrous cellulose crystals on the matrix of *c*-PDTA-DI and the additional crosslinked sites.

**Figure 7:**
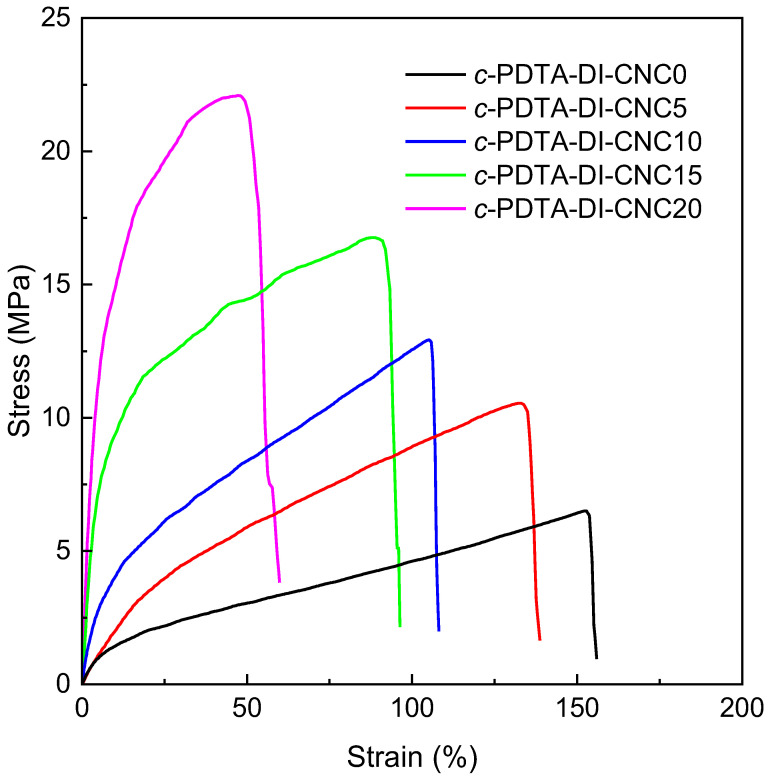
Stress–strain curves of *c*-PDTA-DI-CNC nanocomposites.

For the nanocomposites of *c*-PDTA-DI with CNCs, the mechanical properties were measured through tensile tests; the stress–strain curves are shown in Figure 7. For comparison, the *c*-PDTA networks were also measured under identical conditions; the stress–strain curves are shown in Figure S7. The results of all the mechanical measurements are summarized in Table 2. Of these *c*-PDTA networks, *c*-PDTA-HE displayed the highest Young’s modulus (698 MPa) and tensile strength (43.4 MPa) but the lowest elongation at break (6.2%); *c*-PDTA-DI exhibited the lowest Young’s modulus (21.2 MPa) and tensile strength (7.63 MPa) but the highest elongation at break (122.7%). The difference in mechanical properties is attributed to the difference in the network structures, which influenced the *T*_g_ values and crosslinking densities. The larger the lengths of diol molecules, the lower the crosslinking densities of *c*-PDTA networks, and the lower the *T*_g_’s. As a result, the *c*-PDTA-HE and *c*-PDTA-DI networks displayed the highest and lowest stiffness but the lowest and largest ductility. For the CNC-containing nanocomposites, the stress–strain curves (Figure 7) showed that the incorporation of CNCs significantly enhanced both the Young’s modulus and tensile strength of the networks (Table 3). For instance, the *c*-PDTA-DI-CNC0 displayed the Young’s modulus and tensile strength of 19.5 MPa and 6.4 MPa, respectively. Upon inclusion of CNCs, these properties were significantly enhanced. Notably, the nanocomposite containing 20 wt% CNCs exhibited a Young’s modulus of 236.2 MPa and a tensile strength of 22.13 MPa, representing an increase of more than tenfold in modulus and threefold in tensile strength. Notably, the elongations at break (*ε*_b_’s) decreased with the incorporation of CNCs. The higher the contents of CNCs, the lower the elongations at break. The generation of a great number of interfaces between *c*-PDTA and CNCs is responsible for the decrease in *ε*_b_’s. Similar results have also been found in other polymer nanocomposites reinforced with rigid nano-objects.

### 3.4. Reprocessing Properties

**Figure 8:**
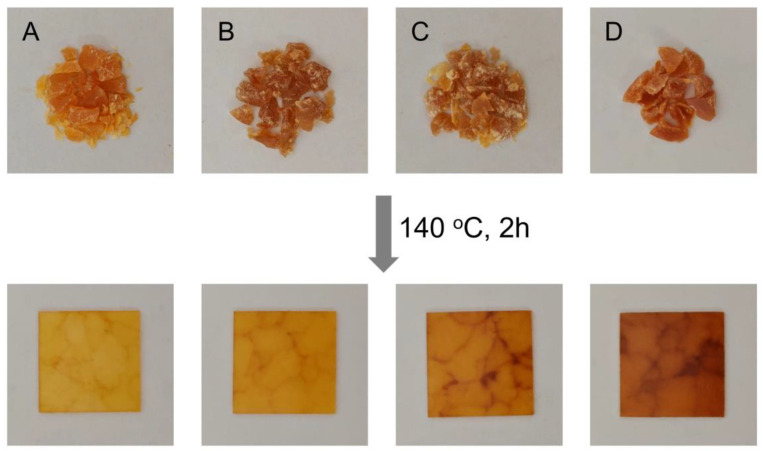
Photographs of (**A**) *c*-PDTA-DI-CNC5, (**B**) *c*-PDTA-DI-CNC10, (**C**) *c*-PDTA-DI-CNC15 and (**D**) *c*-PDTA-DI-CNC20 for reprocessing tests.

**Figure 9:**
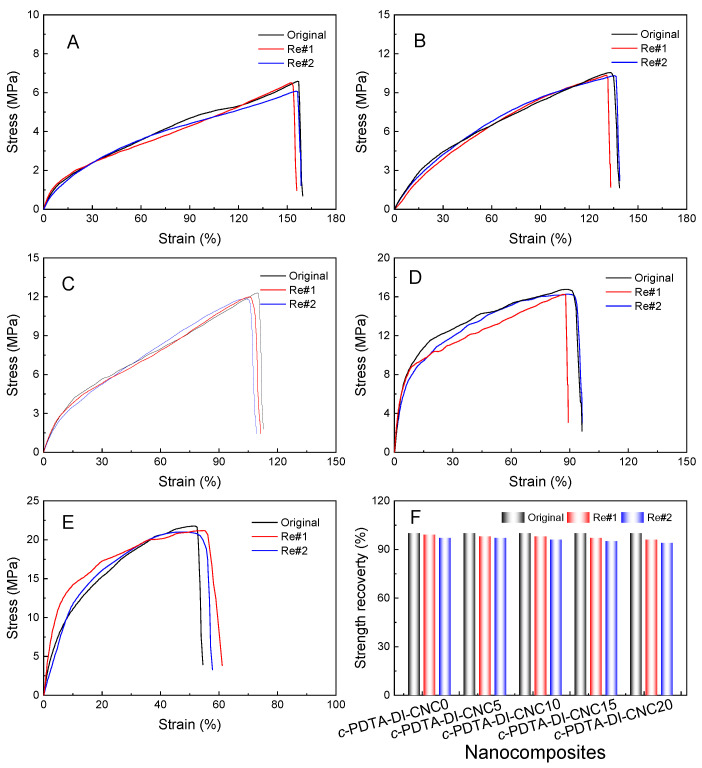
Stress–strain curves of (**A**) *c*-PDTA-DI-CNC0, (**B**) *c*-PDTA-DI-CNC5, (**C**) *c*-PDTA-DI-CNC10, (**D**) *c*-PDTA-DI-CNC15 and (**E**) *c*-PDTA-DI-CNC20 before and after reprocessing. (**F**) Recovery of mechanical strengths of the nanocomposites.

Serving as the dynamic chemistry, the exchange of dithioacetal bonds would impart reprocessing properties to the *c*-PDTA-DI networks. To demonstrate this, the samples were fragmented and then subjected to hot-pressing at 140 °C. Notably, all the samples can be reprocessed into monolithic sheets within 2 h (Figure 8 and Figure S8), demonstrating that the *c*-PDTA-DI networks were indeed malleable. To examine the mechanical strengths of the recycled samples, the stress–strain tests were further performed as shown in Figure 9 and Figure S9. The recycled samples had the stress–strain curves very close to the original after the samples were reprocessed two and three times, showing that the mechanical strengths were not significantly sacrificed with the reprocessing tests. All the *c*-PDTA-DI networks still had 90% (or higher) of the tensile strengths of the original samples, demonstrating excellent reprocessability. It is worth noticing that the incorporation of CNCs did not cause a decrease in the tensile strength for the recycled nanocomposites.

**Figure 10:**
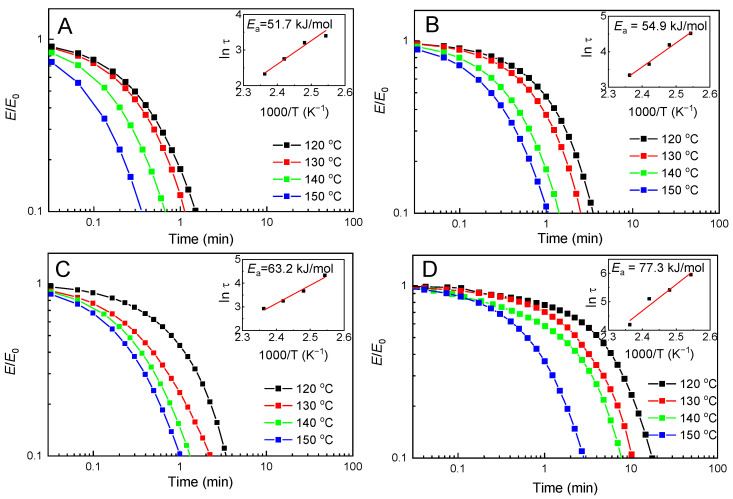
Stress relaxation curves of (**A**) *c*-PDTA-DI, (**B**) *c*-PDTA-TH, (**C**) *c*-PDTA-DE and (**D**) *c*-PDTA-HE.

The dynamic exchange of dithioacetal bonds is responsible for the excellent reprocessing properties. It is of interest to examine the impacts of (i) the types of dithiols and (ii) the incorporation of CNCs on the dynamic chemistries. Toward this end, the tensile stress relaxation tests were carried out at variable temperatures from 120 to 150 °C; the stress relaxation curves are shown in Figure 10. In all of the cases, the stresses can be well relaxed. The higher the temperature, the faster the stress was relaxed. The stress relaxation behavior indicates that the re-curation (or reshuffle) of the networks indeed occurred through the exchange of dithioacetal bonds. If the time at which the stress was relaxed to 1/e of the initial is defined as the time of stress relaxation (*τ*), the *τ* value can be used to estimate the activation energy (*E*_a_) of the exchange of dithioacetal bonds with the Arrhenius model as below [31]:

(2) $$\tau =Ae^{\frac{E_{a}}{RT}}$$

where *A* and *R* are the pre-exponential factor and gas constant. For *c*-PDTA-DI, *c*-PDTA-TH, *c*-PDTA-DE and *c*-PDTA-HE, the *E*_a_’s were 51.7, 54.9, 63.2 and 77.3 kJ × mol^−1^. It was found that the *E*_a_’s were quite dependent on the types of dithiols used. A careful comparison shows that the order of *E*_a_’s from low to high correlates with the lengths of dithiol molecules, which determined the concentration of dithioacetal bonds in the *c*-PDTA networks. The higher the concentration, the lower the *E*_a_’s. It seems that an increase in the concentration of dithioacetal bonds facilitates the exchange of dithioacetal bonds. Nonetheless, the increase in the concentration of dithioacetal bonds would cause an increase in crosslinking densities, which constitutes a factor of enhancing *E*_a_’s. The result that the overall *E*_a_’s were increased indicates that the impact of concentration on crosslinking density was the dominant factor.

**Figure 11:**
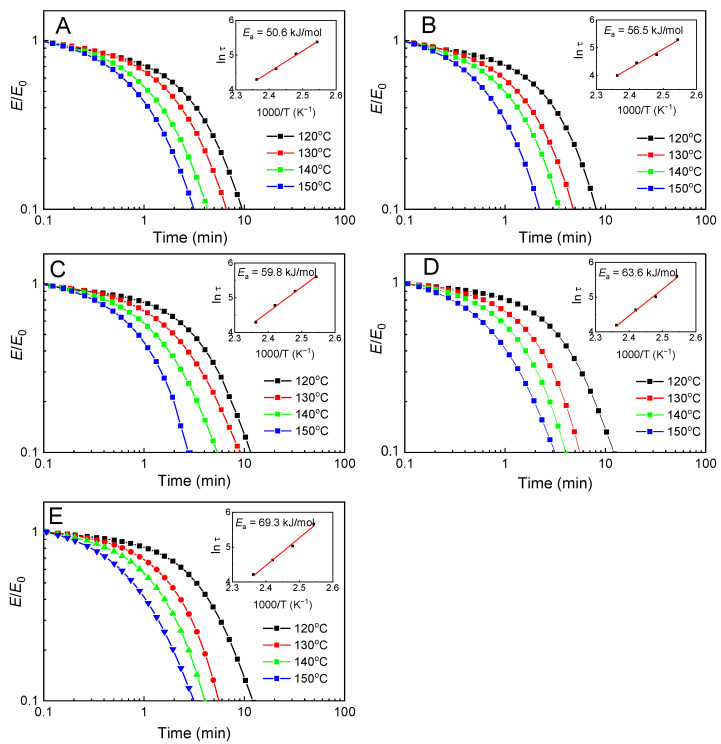
Stress relaxation curves of (**A**) *c*-PDTA-DI-CNC0, (**B**) *c*-PDTA-DI-CNC5, (**C**) *c*-PDTA-DI-CNC10, (**D**) *c*-PDTA-DI-CNC15 and (**E**) *c*-PDTA-DI-CNC20.

Upon introducing CNCs, the *E*_a_’s were increased. Taking *c*-PDTA-DI-CNC, for instance, the *E*_a_’s were measured as shown in Figure 11. By varying the concentration of CNCs from 0 to 20 wt%, the *E*_a_’s were increased from 50.6 to 69.3 kJ × mol^−1^. The increase in *E*_a_ suggests that the difficulty in the exchange of dithioacetal bonds increased with increasing the concentration of CNCs. It is proposed that the fine dispersion of CNCs in the *c*-PDTA-DI matrix would restrict the exchange of dithioacetal bonds. The higher the concentration of CNCs, the stronger the restriction. Nonetheless, the *E*_a_ values were quite low (*viz*. 50.6 to 69.3 kJ × mol^−1^), indicating that the restriction was not very strong. As a result, the nanocomposites still displayed excellent reprocessing properties.

## 4. Conclusions

In summary, biomass-based nanocomposites of polydithioacetals with cellulose nanocrystals were successfully synthesized via the co-crosslinking of linear polydithioacetals, surface-functionalized CNCs and diglycidyl ether of bisphenol A. It was found that CNCs were finely dispersed in the networks of polydithioacetals. The incorporation of CNCs resulted in the nanocomposites displaying improved thermomechanical properties. Compared to the network without CNCs, the nanocomposite with 20 wt% of CNCs exhibited a tenfold increase in Young’s modulus and a threefold increase in tensile strength. Benefiting from the integration of dynamic dithioacetal bonds, the nanocomposites displayed excellent reprocessing properties. The dynamic exchange of dithioacetal bonds is responsible for the reprocessing behavior. This work presents a promising strategy for developing bio-based nanocomposites that not only have improved thermomechanical properties but also excellent reprocessing (or recycling) properties.

## Figures and Tables

**Table 1:** Molecular weights of linear PDTAs.

PDTAs	Dithiols	*M*_n_ (Da)	*M*_w_/*M*_n_
PDTA-HE	1,6-hexanedithiol,	42,200	1.34
PDTA-DE	1,10-decanedithiol,	38,600	1.32
PDTA-TH	2,2′-thiodiethanethiol	34,200	1.40
PDTA-DI	3,6-dioxa-1,8-octanedithiol	36,700	1.36

**Table 2:** Thermal and mechanical properties of *c*-PDTA networks.

Samples	*T*_g_ (°C)	*σ*_b_(MPa)	*E* (MPa)	*ε*_b_ (%)
*c*-PDTA-HE	48.0	43.40 ± 4.51	698 ± 10.4	6.2 ± 0.6
*c*-PDTA-DE	37.8	30.62 ± 3.26	556 ± 8.9	7.6 ± 1.5
*c*-PDTA-TH	33.6	10.03 ± 2.08	360 ± 5.7	47.2 ± 2.7
*c*-PDTA-DI	27.8	7.63 ± 1.45	21.2 ± 2.5	122.7 ± 5.8

**Table 3:** Thermal and mechanical properties of *c*-PDTA-DI-CNC nanocomposites.

Samples	*T*_g_ (°C)	*σ*_b_ (MPa)	*E* (MPa)	*ε*_b_ (%)
*c*-PDTA-DI-CNC0	20.9	6.40 ± 0.32	19.5 ± 2.2	153.6 ± 8.4
*c*-PDTA-DI-CNC5	22.1	10.45 ± 1.03	21.2 ± 3.1	133.3 ± 6.9
*c*-PDTA-DI-CNC10	25.2	12.92 ± 1.21	112.3 ± 2.3	106.7 ± 5.4
*c*-PDTA-DI-CNC15	28.6	16.75 ± 1.91	156.5 ± 3.8	91.6 ± 5.1
*c*-PDTA-DI-CNC20	32.7	22.13 ± 2.17	236.2 ± 5.1	48.2 ± 2.8

## Data Availability

Data are contained within the article and Supplementary Materials.

## Supplementary Materials

The following supporting information can be downloaded at: https://www.mdpi.com/article/10.3390/polym17131764/s1, Figure S1. FTIR spectra of vanillin, 3,6-dioxa-1,8-octanedithiol and PDTA-DI.; Figure S2: FTIR spectra of CNCs and thiol-functionalized CNCs; Figure S3: TGA curves of CNCs and thiol-functionalized CNCs; Figure S4: FTIR spectra of *c*-PDTA-HE, *c*-PDTA-DE, *c*-PDTA-TH and *c*-PDTA-DI networks; Figure S5: Gel fractions of *c*-PDTA-HE, *c*-PDTA-DE, *c*-PDTA-TH and *c*-PDTA-DI in THF at room temperature; Figure S6: Rheological frequency sweeps of (A) *c*-PDTA-DI-CNC0, (B) *c*-PDTA-DI-CNC5 and (C) *c*-PDTA-DI-CNC15; Figure S7: Stress–strain curves of *c*-PTDA networks; Figure S8: Reprocessing photos of (A) *c*-PDTA-HE, (B) *c*-PDTA-DE, (C) *c*-PDTA -TH and (D) *c*-PDTA-DI; Figure S9: Stress–strain curves of the networks before and after reprocessing: (A) *c*-PDTA-HE, (B) *c*-PDTA-DE, (C) *c*-PDTA-TH and (D) *c*-PDTA-DI.
