# Supplementary material for: Biomass-Based Nanocomposites of Polydithioacetals Derived from Vanillin with Cellulose Nanocrystals: Synthesis, Thermomechanical and Reprocessing Properties
Source: Polymers (Basel). 2025 Jun 26;17(13):1764. doi: 10.3390/polym17131764 (PMC12251680; doi:10.3390/polym17131764)
Supplement: Supplementary file 1 [file polymers-17-01764-s001.zip › polymers-3679222-supplementary.pdf]

SUPPORTING INFORMATION for

Biomass-based Nanocomposites of Polydithioacetals Derived from Vanillin and Cellulose Nanocrystals: Synthesis, Thermomechanical and Reprocessing Properties

CONTENTS

1. Measurements and Techniques……………………………………………S3

1.1. Nuclear Magnetic Resonance (NMR) Spectroscopy………………………… S3

1.2. Gel Permeation Chromatography (GPC) ……………………..……………S3

1.3. Fourier Transform Infrared (FTIR) Spectroscopy…………………………S3

1.4. Thermal Gravimetric Analysis (TGA)………………………...................S3

1.5. Differential Scanning Calorimetry (DSC) ………………………………S3

1.6. Stress Relaxation Measurements ……………………………………S3

1.7. Tensile Mechanical Tests………………………………………………S4

1.8. Rheological Measurements…………………………………………………S4

1.9. Transmission Electron Microscopy (TEM) …………………………………S4

2. Synthesis of PTDAs……………………………………………………………S5

3. Figures ………………………………………………………………………S6

1. Measurements and Techniques

Nuclear magnetic resonance (NMR) spectroscopy

^{1}H NMR measurements were performed on a Bruker Advance 500 spectrometer at room temperature. Deuterium chloroform was used as the deuterated solvent.

Gel Permeation Chromatography (GPC)

The gel permeation chromatography (GPC) analyses were performed using a Waters 1515 HPLC system with an RI detector, along with three Waters RH columns (RH1, 3, 4). For elution, a solution of N,N dimethylformamide (DMF) containing 0.01 M lithium bromide (LiBr) was employed at a constant flow rate of 1.0 mL × min^{-1}. The molar mass values were determined in relation to polystyrene standards.

Fourier transform infrared (FTIR) Spectroscopy

Fourier transform infrared (FTIR) measurements were conducted on a Perkin-Elmer Paragon 100 spectrometer from 4000 to 400 cm^{-1}. The films of specimens were obtained by casting the THF solutions of the samples onto KBr windows. The thermosetting blends were compressed into ultrathin transparent films on a hot press at 130 °C with a pressure of 10MPa for 30 min. The as-obtained ultrathin transparent films were directly measured.

Thermal gravimetric analysis (TGA)

Thermogravimetry analysis (TGA) was performed on a Discovery TGA550 instrument in the temperature range of 50 ~ 800 °C at 20 °C/min under an air atmosphere.

Differential scanning calorimetry (DSC)

Thermal analysis was performed on a TA Instruments Q2000 differential scanning calorimeter in a dry nitrogen atmosphere. The instrument was calibrated with a standard indium.

Stress Relaxation Measurements

Stress relaxation tests were performed with a TA Q800 dynamic mechanical thermal analyzer. The measurements were performed with a constant strain of 5 % at different temperatures.

Tensile Mechanical Tests

Tensile mechanical tests were performed with a WDW-2 electron universal testing machine (Songdun Instruments Co. Ltd., Shanghai, China). The specimens, with dimensions of 40 × 4.5 × 1 mm^3, were prepared in accordance with the ASTM D638 standard. The uniaxial stretching experiments were carried out at a loading rate of 100 mm/min at room temperature. For each sample, five parallel specimens were tested, and then the average results were reported.

Rheological Measurements

The rheological measurements were performed on a DHR-2 stress-controlled rheometer (TA, USA) at 100 °C. The linear viscoelastic (LVE) regime of each circular specimen was determined by applying strain sweeps at a constant frequency of 1Hz.

Transmission Electron Microscopy (TEM)

Transmission electron microscopy (TEM) was performed on a JEOL JEM-2010 high-resolution transmission electron microscope at an acceleration voltage of 120 kV. The samples were first frozen and ground into powders in liquid nitrogen. The as-obtained powders were then dispersed in ethanol and dropped on the copper grids. After evaporating the solvent, the morphologies of the samples were observed.

Synthesis of PTDAs

Typically, PDTA-DI (6.012 g), DGEBA (3.528 g) and tetrabutylammonium bromide (TBAB) (0.286 g) were dissolved in 1,4-dioxane (10 mL). The mixture was heated at 120 ºC for 10 hours, during which it was gradually gelled. After the solvent was removed under vacuum at 40 ºC, the crosslinked network, denoted *c*-PDTA-DI, was obtained. With a similar procedure, the crosslinked networks containing 1,6-hexanedithiol, 1,10-decanedithiol and 2,2'-thiodiethanethiol were synthesized and denoted *c*-PDTA-HE, *c*-PDTA-DE- and *c*-PDTA-TH, respectively.

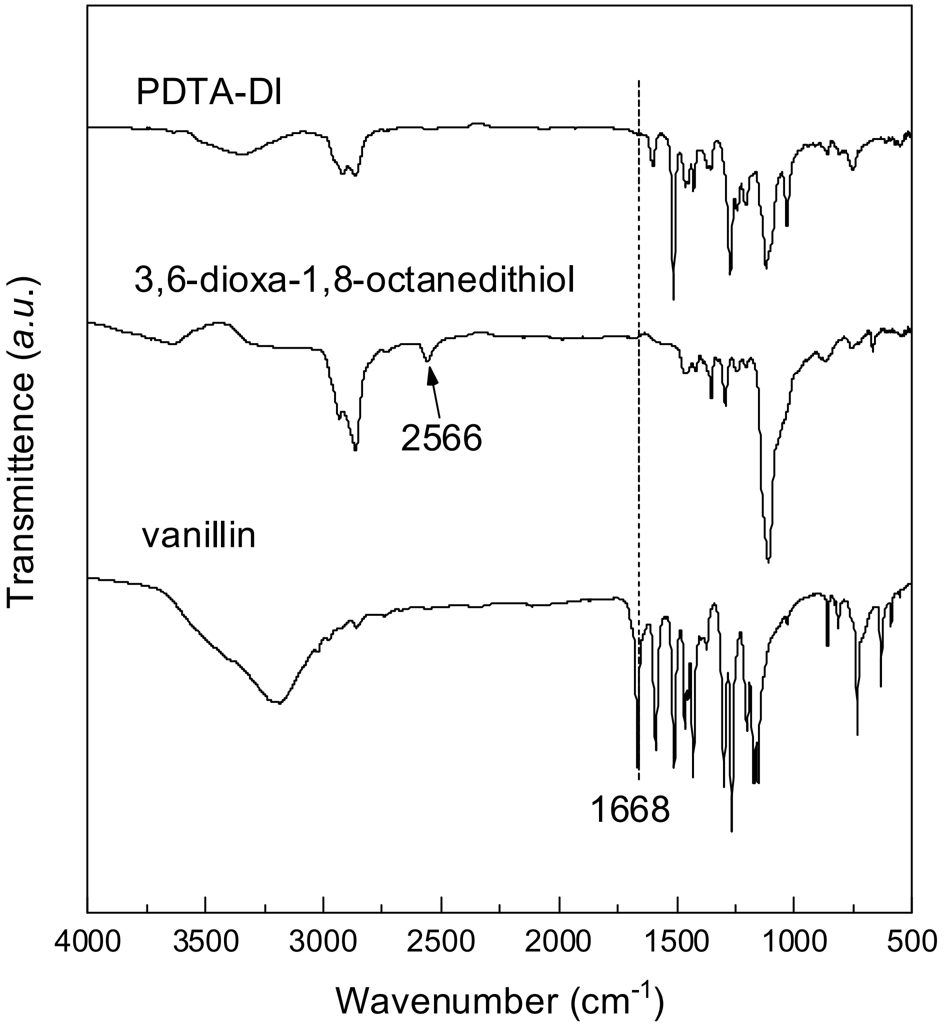

Figure S1. FTIR spectra of vanillin, 3,6-dioxa-1,8-octanedithiol and PDTA-DI

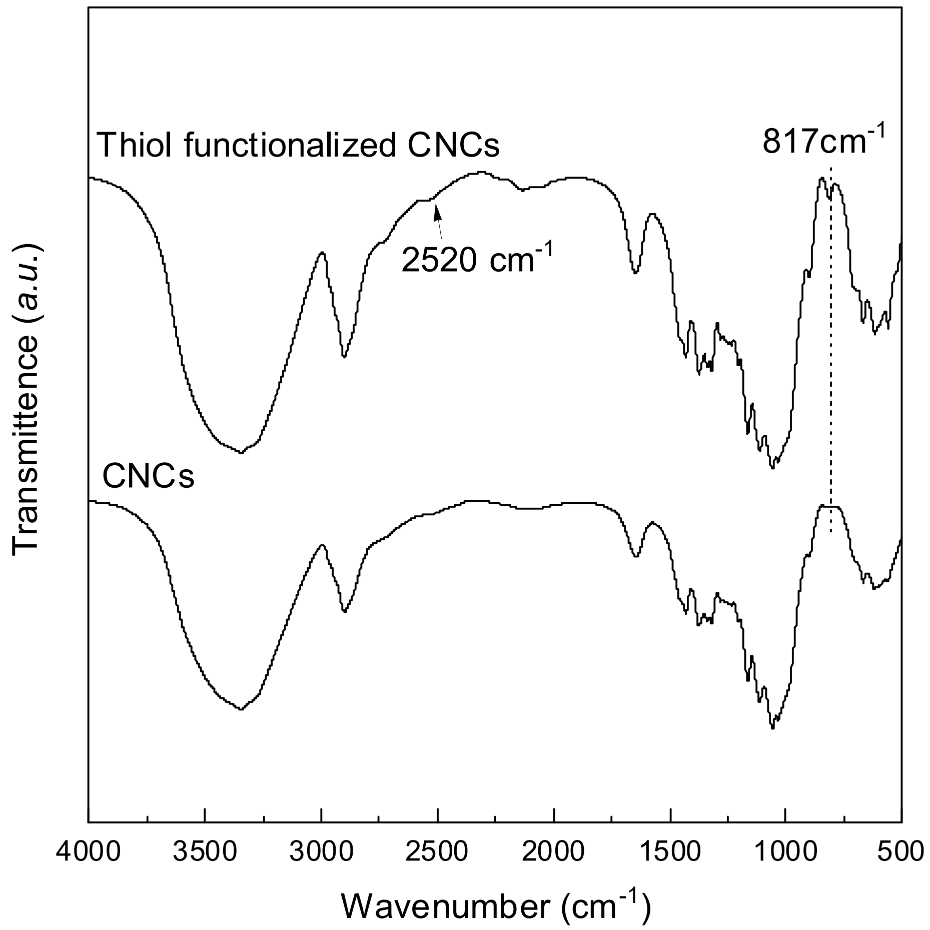

Figure S2. FTIR spectra of CNCs and thiol-functionalized CNCs

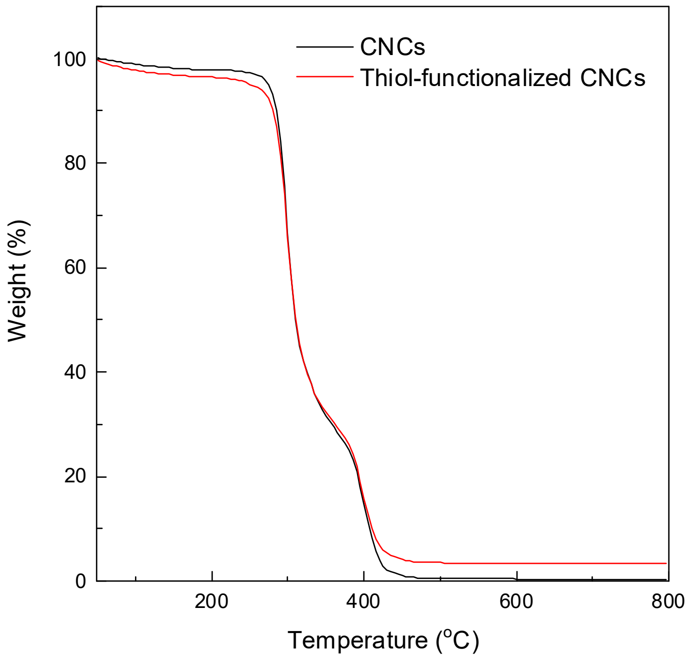

Figure S3. TGA curves of CNCs and thiol-functionalized CNCs.

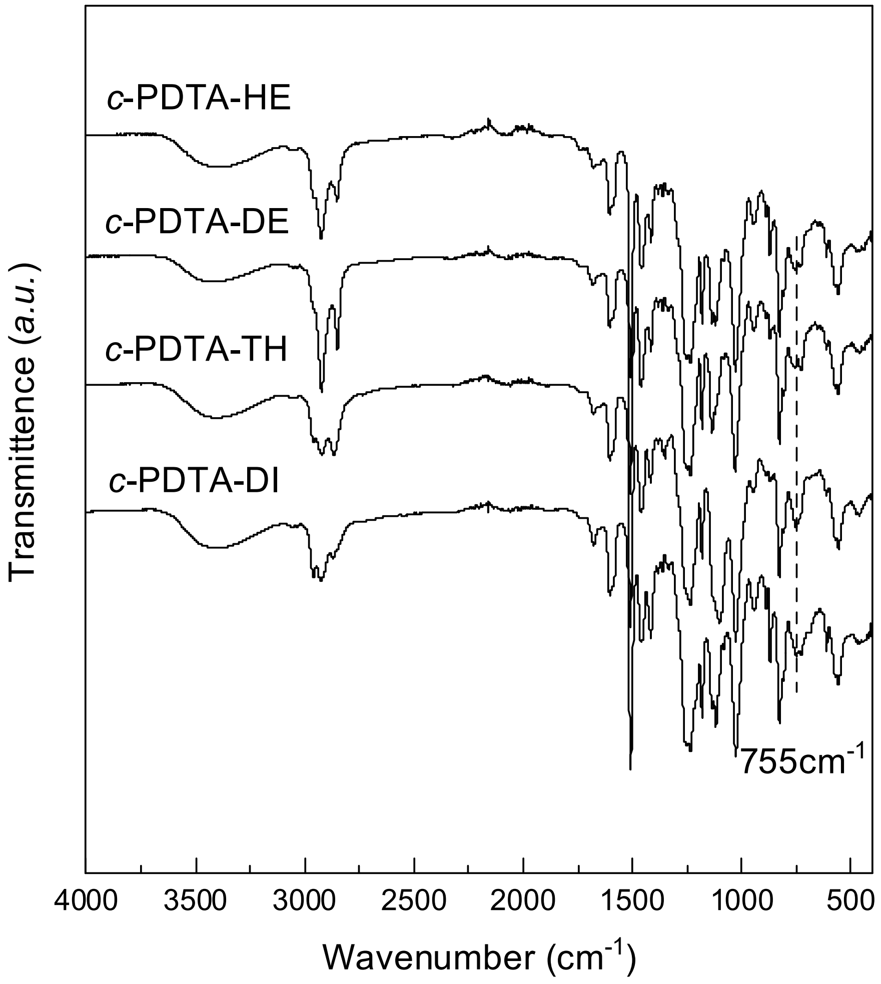

-

Figure S4. FTIR spectra of *c*-PDTA-HE, *c*-PDTA-DE, *c*-PDTA-TH and *c*-PDTA-DI networks.

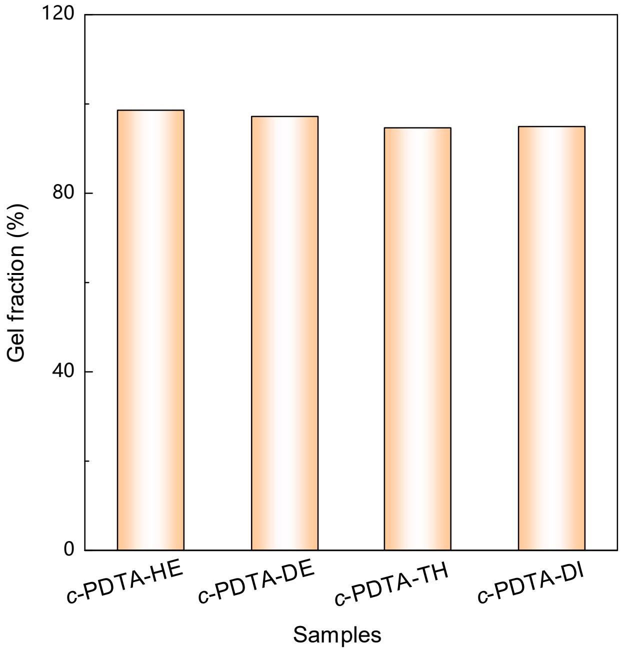

Figure S5. Gel fractions of *c*-PDTA-HE, *c*-PDTA-DE, *c*-PDTA-TH and *c*-PDTA-DI in THF at room temperature

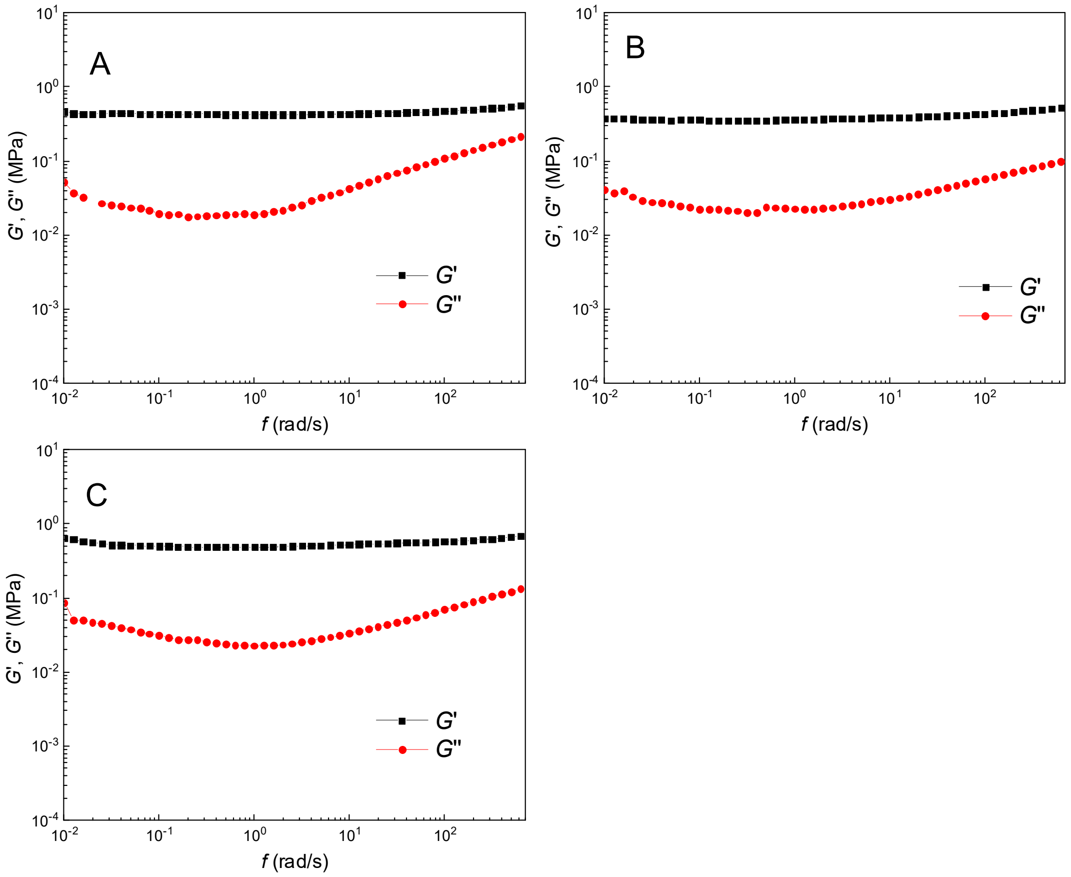

Figure S6. Rheological frequency sweeps of (A) *c*-PDTA-DI-CNC0, (B) *c*-PDTA-DI-CNC5 and (C) *c*-PDTA-DI-CNC15

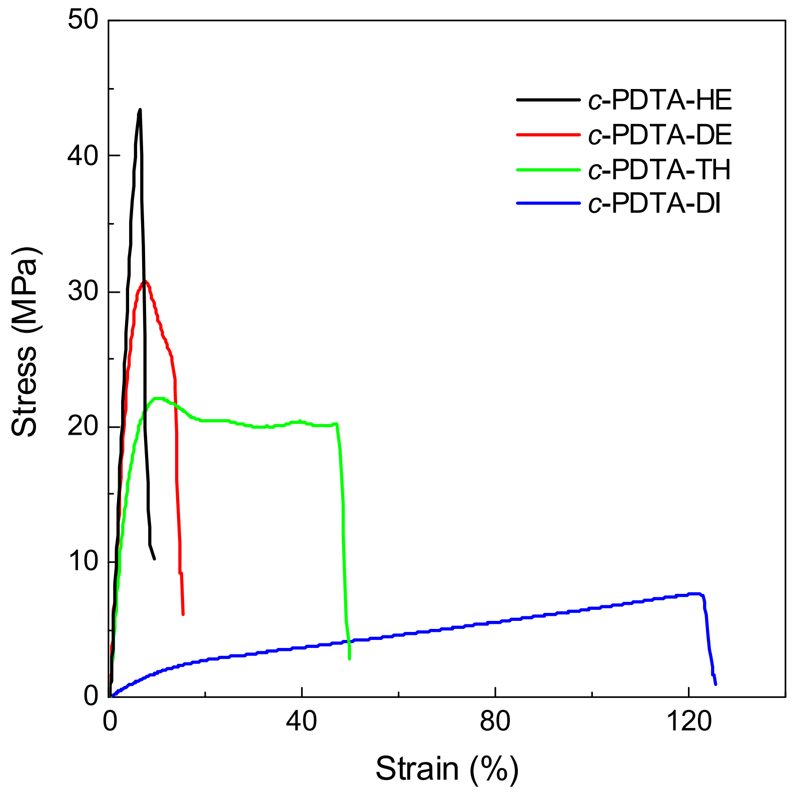

Figure S7. Stress–strain curves of *c*-PDTA networks

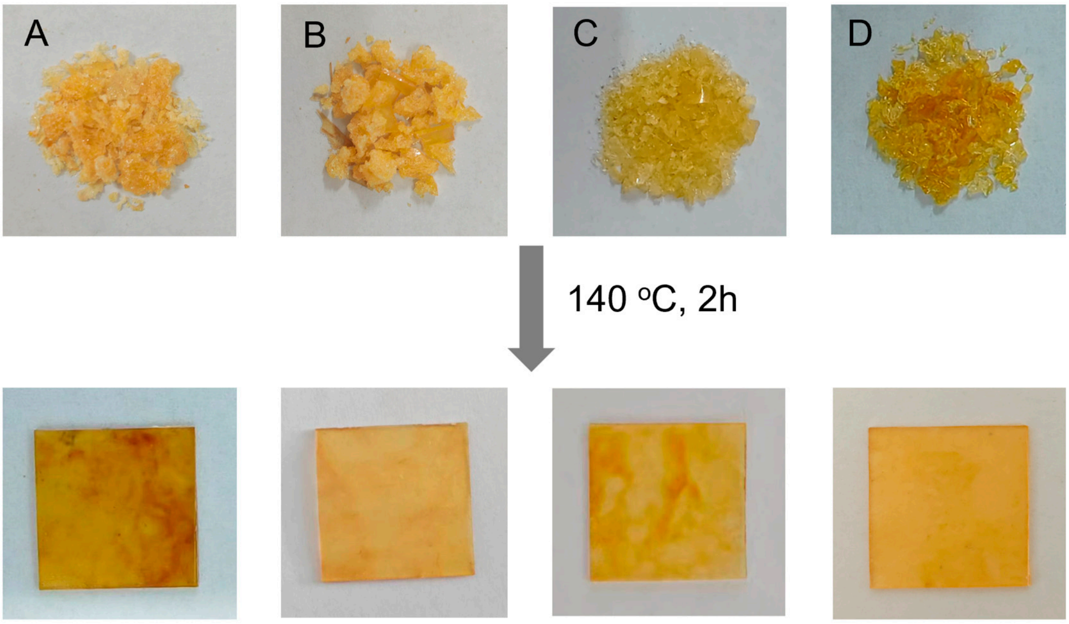

Figure S8. Reprocessing photos of (A) *c*-PDTA-HE, (B) *c*-PDTA-DE, (C) *c*-PDTA - TH and (D) *c*-PDTA-DI

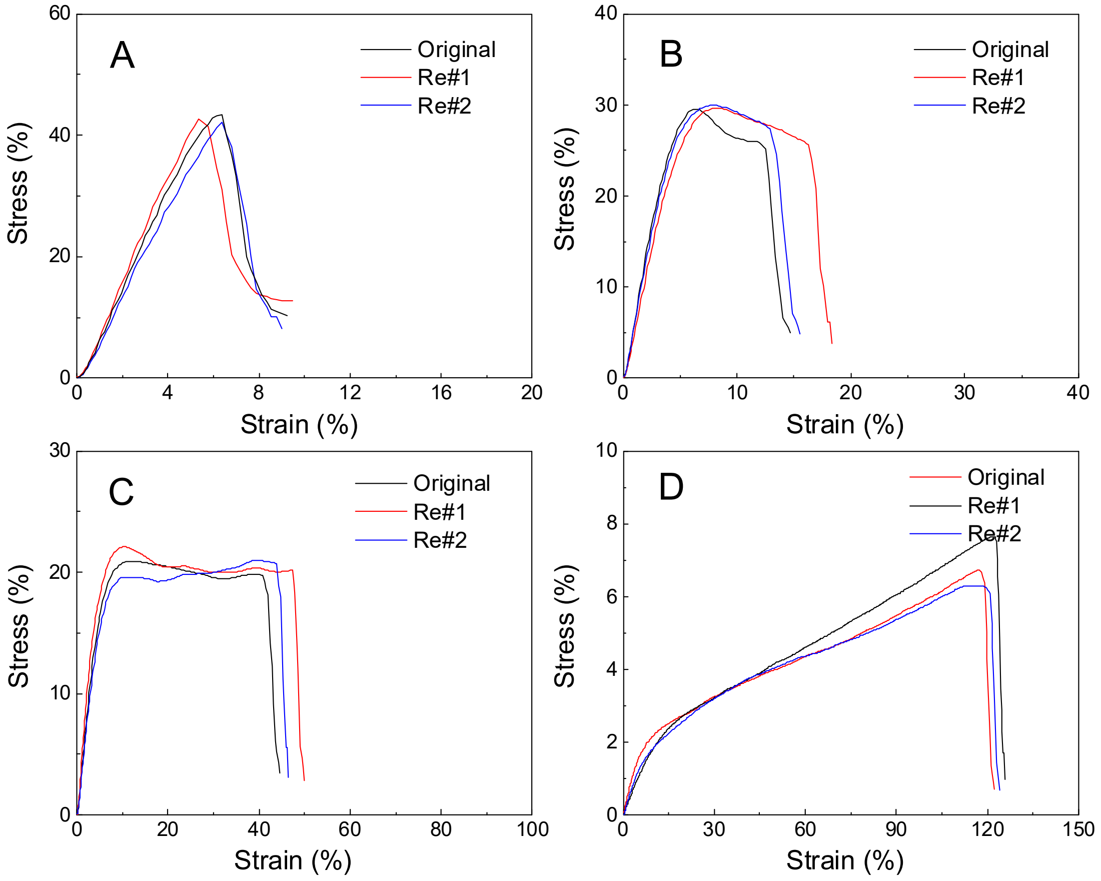

Figure S9. Stress–strain curves of the networks before and after reprocessing: (A) *c*-PDTA-HE, (B) *c*-PDTA-DE, (C) *c*-PDTA-TH and (D) *c*-PDTA-DI.
